# Computational Model of the Effect of Mitochondrial Dysfunction on Excitation–Contraction Coupling in Skeletal Muscle

**DOI:** 10.1007/s11538-022-01079-3

**Published:** 2022-09-17

**Authors:** Sageanne Senneff, Madeleine M. Lowery

**Affiliations:** School of Electrical and Electronic Engineering, University College Dublin, Belfield, Dublin 4, Ireland

**Keywords:** Mathematical modeling, Calcium dynamics, Force, Apoptosis, Muscle weakness

## Abstract

It has become well established that mitochondria not only regulate myoplasmic calcium in skeletal muscle, but also use that calcium to stimulate oxidative phosphorylation (OXPHOS). While experimental approaches have allowed for imaging of mitochondrial calcium and membrane potentials in isolated fibers, capturing the role of mitochondria and the impact of mitochondrial impairments on excitation–contraction coupling (ECC) remains difficult to explore in intact muscle. Computational models have been widely used to examine the structure and function of skeletal muscle contraction; however, models of ECC to date lack communication between the myoplasm and mitochondria for regulating calcium and ATP during sustained contractions. To address this, a mathematical model of mitochondrial calcium handling and OXPHOS was integrated into a physiological model of ECC incorporating action potential propagation, calcium handling between the sarcoplasmic reticulum (SR) and the myoplasm, and crossbridge cycling. The model was used to examine the protective role of mitochondria during repeated stimulation and the impact of mitochondrial dysfunction on ECC resulting from progressive OXPHOS inhibition. Pathological myoplasmic calcium accumulation occurred through distinct mechanisms in the model in the case of either electron transport chain, F1F0 ATP synthase, or adenine nucleotide transporter impairments. To investigate the effect of each impairment on force, a model of calcium-stimulated apoptosis was utilized to capture dysfunction-induced reductions in muscle mass, driving whole muscle force loss. The model presented in this study can be used to examine the role of mitochondria in the regulation of calcium, ATP, and force generation during voluntary contraction.

**Supplementary Information:**

The online version contains supplementary material available at 10.1007/s11538-022-01079-3.

## Introduction

Experimental studies have demonstrated that mitochondria play a key role in modulating myoplasmic calcium during both twitch and tetanic contractions via calcium uptake mechanisms (Rudolf et al. 2004; Shkryl and Shirokova 2006; Yi et al. 2011), stimulating oxidative phosphorylation (OXPHOS) in skeletal muscle (Glancy and Balaban 2012; Glancy et al. 2013; Pan et al. 2013). Evidence of skeletal muscle mitochondrial dysfunction has been observed in neurodegenerative and muscular disorders with both humans and animal models, including Parkinson’s disease (Winkler-Stuck et al. 2005; Shtifman et al. 2011; Kelly et al. 2014), Huntington’s disease (Lodi et al. 2000; Ciammola et al. 2006), amyotrophic lateral sclerosis (Dobrowolny et al. 2008), and muscular dystrophy (Millay et al. 2008; Turki et al. 2012). Mitochondrial dysfunction in skeletal muscle has been attributed to genetic mutations involved in OXPHOS from human data (Waragai et al. 2007; Gdynia et al. 2009; Jin and Johnson 2010) and observed in genetic knockout models in fruit flies (Park et al. 2006) and mice (Shtifman et al. 2011).

Mitochondrial abnormalities resulting in a loss of muscle mass have been linked to disruptions in calcium homeostasis (Romanello and Sandri 2016) and the mitochondrial membrane potential (Zorova et al. 2018), as well as upregulation of apoptotic factors p53 and active caspase-3, correlated with reduced muscle fiber area (Erekat 2015). However, mitochondrial deficiencies do not present across all subjects in human pathologies (Lodi et al. 2000; Kelly et al. 2014), and the impact of peripheral mitochondrial impairments on myoplasmic calcium, ATP, and force generation is difficult to explore experimentally in vivo. In this context, computational modeling can provide a powerful tool to investigate the role of mitochondria during excitation–contraction coupling (ECC) and consequences of dysfunction.

While earlier studies have combined experimental and computational approaches to monitor and model the kinetics of ATP production in skeletal muscle fibers (Korzeniewski and Zoladz 2001; Wu et al. 2007; Korzeniewski 2015; Matsuda et al. 2020), these models do not incorporate functional dependence of mitochondria on calcium. Conversely, models that track calcium exchange between the sarcoplasmic reticulum (SR), myoplasm, and mitochondria neglect OXPHOS (Groenendaal et al. 2008; Marcucci et Al. 2018). A more recent model of ECC incorporating mitochondrial metabolism was used to explore the impact of metabolic energy systems on crossbridge cycling (Wang et al. 2020); however, the link between calcium and OXPHOS was still missing.

To address these gaps, a model of mitochondrial calcium handling and calcium-activated OXPHOS was integrated into a model of ECC in single muscle fibers developed previously by the authors (Senneff and Lowery 2021), which included a propagating ionic action potential, calcium handling between the SR and myoplasm, and crossbridge cycling to generate force. The combined model presented here was able to demonstrate the role of mitochondria in regulating myoplasmic calcium during repetitive stimulation at increasing frequencies. The model was then used to examine the effect of OXPHOS impairments on ECC during tetanic stimulation by inhibiting: (1), the electron transport chain, responsible for NADH oxidation, (2), the F1F0 ATP synthase, responsible for mitochondrial ATP synthesis, and (3), the adenine nucleotide transporter, responsible for exchanging mitochondrial ATP and myoplasmic ADP. These three model components were selected for their pathological associations with Parkinson’s disease (Winkler-Stuck et al. 2005; Kelly et al. 2014), Huntington’s disease (Lodi et al. 2000), and muscular dystrophy (Turki et al. 2012), respectively. Finally, whole muscle force loss in response to OXPHOS inhibition was captured using a model of calcium-stimulated apoptosis, emulating muscle weakness during sustained contractions as a result of reduced muscle mass.

## Methods

### Model Design

**Fig. 1:**
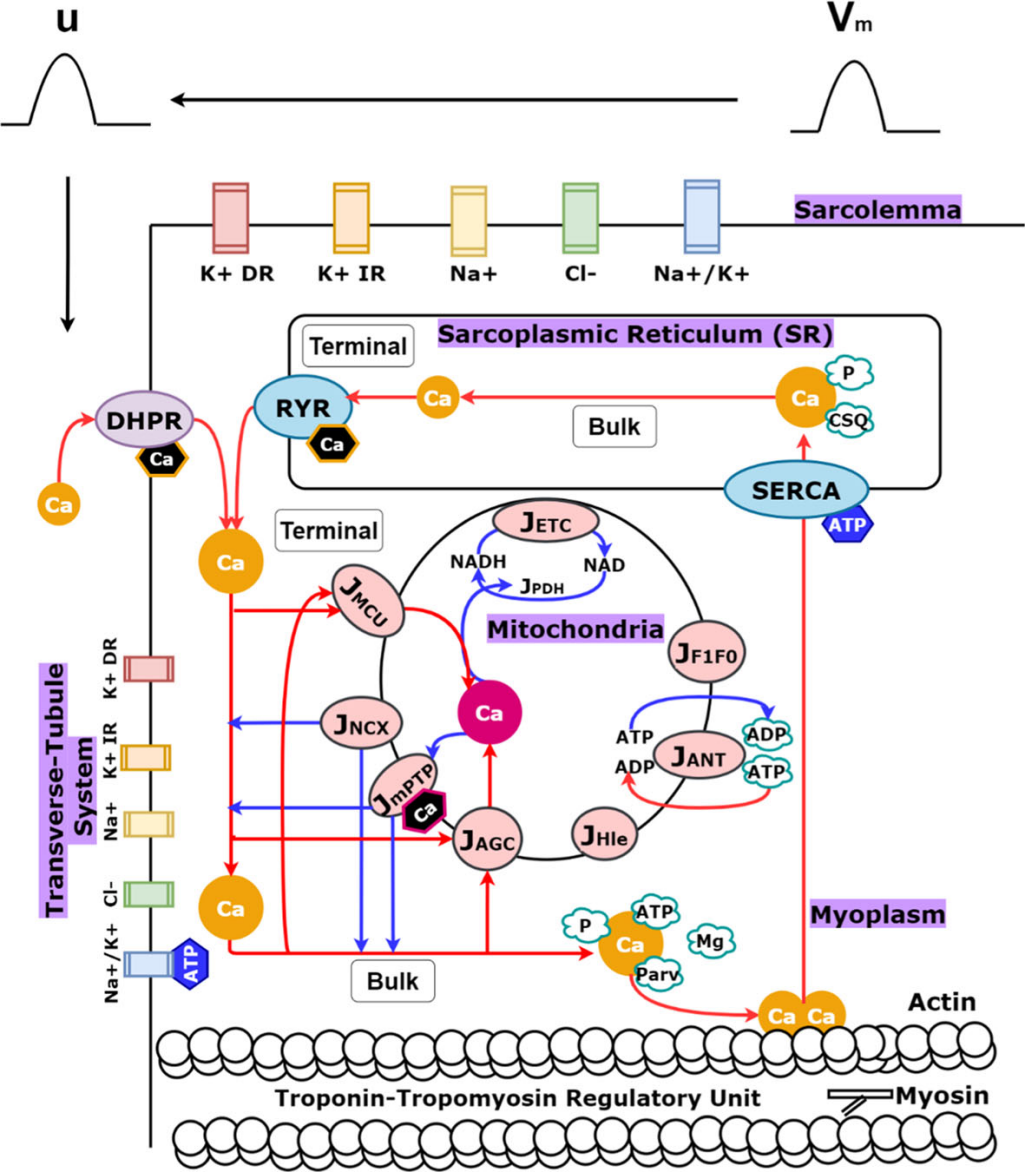
(Color figure online) Model schematic. Four ionic channels (labeled K + DR, K + IR, Na+, Cl−) and a pump (labeled Na+/K+) were placed in the sarcolemma compartment to generate an action potential (labeled ${V}_{\mathrm{m}}$). Five ionic channels [labeled DHPR (generating an L-type calcium channel), K + DR, K + IR, Na+, Cl−] and a pump (labeled Na+/K+) were placed in the transverse tubular system compartment to generate an action potential (labeled $u$). Half-sarcomere components in the transverse tubular system are visualized (labeled sarcoplasmic reticulum, myoplasm, mitochondria) along with indications of their further spatial distinctions (labeled terminal or bulk). The terminal and bulk mitochondria are modeled identically, except for their spatial myoplasmic dependencies (i.e., terminal mitochondrial calcium uptake relies on terminal myoplasmic calcium concentration, while bulk mitochondrial calcium uptake relies on bulk myoplasmic calcium concentration, and so on). Blue arrows indicate movement from mitochondria to the myoplasm, while red arrows indicate the reverse, as well as other ECC processes. Calcium binding to troponin to initiate crossbridge cycling via actin–myosin interactions is pictured. Hexagonal shapes indicate points of feedback in the model from calcium or ATP. Mitochondrial calcium dependence is highlighted in pink, while myoplasmic calcium dependence is highlighted in orange. Myoplasmic ATP dependence is highlighted in blue

A single muscle fiber was modeled with two compartments, one representing the sarcolemma and one representing the transverse tubular system, as previously described in Senneff and Lowery (2021). The sarcolemma and transverse tubular compartments each contained a delayed-rectifier potassium channel, an inward rectifier–potassium channel, a sodium channel, a chloride channel, and a sodium–potassium pump, incorporated at varying densities per compartment. The transverse tubular compartment additionally contained a voltage and calcium-dependent L-type calcium channel. A half-sarcomere was placed into the transverse tubular system which contained three further sub-compartments: the sarcoplasmic reticulum (SR), myoplasm, and mitochondria (Fig. 1).

The SR sub-compartment includes calcium release and uptake machinery, as well as a pool of calsequestrin and phosphate for calcium buffering. The myoplasm sub-compartment includes calcium buffers magnesium and parvalbumin, as well as phosphate, ATP, and ADP ions, and the crossbridge machinery required for force generation in the model. The mitochondria sub-compartment includes nine fluxes, derived from a minimal pancreatic beta-cell model (Magnus and Keizer 1998a, b; Bertram et al. 2006; Wacquier et al. 2016) and modified, where possible, for muscle using equations and parameters from computational studies in both cardiac (Cortassa et al. 2003) and skeletal muscle (Korzeniewski and Zoladz 2001; Wu et al. 2007), or otherwise extended as described in the following sections.

In line with previous modeling work to capture localized calcium changes close to the SR calcium release site (Shorten et al. 2007; Senneff and Lowery 2021), each of these three sub-compartments were further split into a ‘terminal’ space closer to the release site and a ‘bulk’ space farther from the release site. Half-sarcomere volume was distributed such that the terminal SR (${T}_{\mathrm{SR}}$) and bulk SR ($\mathrm{SR})$ comprised 5% of the total volume, the terminal mitochondria (${M}_{\mathrm{TM}}$) and bulk mitochondria (${M}_{\mathrm{M}}$) comprised 15.9%, and the remaining 79.1% was comprised of the terminal myoplasm ($\mathrm{TM}$) and bulk myoplasm ($M$). Mitochondria density was computed for a slow twitch muscle fiber (Jackman and Willis 1996; Scorzeto et al. 2013; Pietrangelo et al. 2015). Equations for the entire integrated model are outlined in the following sections, or otherwise presented in Supplementary Material A where indicated. Mitochondria model parameters are presented in Supplementary Material B. All model equations and parameters describing the multi-compartmental model of ECC are described in detail in Senneff and Lowery (2021). The model presented here utilizes model equations and parameters describing slow twitch muscle as given in Senneff and Lowery (2021).

### Action Potential Generation

Action potentials in the sarcolemma compartment were generated with the following equation:

1 $$\frac{\mathrm{d}{V}_{m}}{\mathrm{d}t}= \frac{1}{{C}_{\mathrm{m}}}\left[-{I}_{\mathrm{ionic}}-{I}_{\mathrm{T}}\right]$$

where $\frac{{\mathrm{dV}}_{\mathrm{m}}}{\mathrm{d}t}$ is the rate of change of the membrane potential (${V}_{\mathrm{m}}$), ${C}_{\mathrm{m}}$ is the membrane capacitance, and ${I}_{\mathrm{T}}$ is the total transverse tubule membrane current density. ${I}_{\mathrm{ionic}}$ is the sum of the delayed and inward rectifier potassium, sodium, and chloride currents and sodium–potassium pump current along the sarcolemma. Action potentials in the transverse tubular compartment were generated with the following equation:

2 $$\frac{\mathrm{d}u}{\mathrm{d}t}= \frac{1}{{C}_{\mathrm{m}}}\left[{\frac{{g}_{\mathrm{L}}}{{A}_{\mathrm{T}}}I}_{\mathrm{T}}-{I}_{{\mathrm{ionic}}_{\mathrm{T}}}\right]$$

where $\frac{\mathrm{d}u}{\mathrm{d}t}$ is the rate of change of the membrane potential ($u$). Current density, ${I}_{\mathrm{T}}$, was scaled by the surface area of the muscle fiber, ${A}_{\mathrm{T}}$, and its radial conductivity, ${g}_{\mathrm{L}}$. ${I}_{{\mathrm{ionic}}_{\mathrm{T}}}$ represents the sum of the currents within the transverse tubular system, which are identical to those along the sarcolemma, except for their channel density, and for the inclusion of an L-type calcium current. All model equations and parameters governing muscle fiber geometry and excitation are described in detail in Senneff and Lowery (2021).

### Mitochondrial Membrane Potential

The mitochondrial membrane potential ($\Delta \Psi$) is the driving force for the majority of mitochondrial processes in the model (Wacquier et al 2016):

3 $$\frac{\mathrm{d}\Delta {\Psi }_{{\mathrm{M}}_{x}}}{\mathrm{d}t}= \frac{1}{{C}_{\mathrm{p}}}\left({a}_{1}{J}_{{\mathrm{ETC}}_{{\mathrm{M}}_{x}}} -{a}_{2}{J}_{F1F{0}_{{\mathrm{M}}_{x}}} -{J}_{{\mathrm{ANT}}_{{\mathrm{M}}_{x}}}-{J}_{H,l{e}_{{\mathrm{M}}_{x}}}-{J}_{{\mathrm{NCX}}_{{\mathrm{M}}_{x}}}-2{J}_{{\mathrm{MCU}}_{{\mathrm{M}}_{x}}}-2{J}_{m{\mathrm{PTP}}_{{\mathrm{M}}_{x}}} -{J}_{{\mathrm{AGC}}_{{\mathrm{M}}_{x}}}\right)\quad x \epsilon \left\{\mathrm{TM}, M\right\}$$

tracked within both the terminal mitochondria ($${M}_{x}, x=\mathrm{TM})$$ and bulk mitochondria ($${M}_{x}, x=M)$$ spaces. $${C}_{\mathrm{p}}$$ is a constant representing the mitochondrial membrane capacitance, scaled by Faraday’s constant. The flux through complexes I, III, and IV of the electron transport chain (ETC) is represented by $${J}_{\mathrm{ETC}}$$, scaled by $${a}_{1}$$ representing the change in $$\Delta \Psi $$ due to proton flux in response to NADH production. The flux through the F1F0 ATP Synthase (F1F0) is represented by $${J}_{\mathrm{F}1\mathrm{F}0}$$, scaled by $${a}_{2}$$, representing the change in $$\Delta \Psi $$ in response to ATP production, and the flux through the adenine nucleotide transporter (ANT) is represented by $${J}_{\mathrm{ANT}}$$. A proton leak flux is represented by $${J}_{H,le}$$. Calcium dynamics are captured by $${J}_{\mathrm{MCU}}$$, the flux through the mitochondrial calcium uniporter (MCU), $${J}_{\mathrm{NCX}}$$, the flux through the sodium–calcium exchanger, and $${J}_{\mathrm{mPTP}}$$, the flux through the mitochondrial permeability transition pore (mPTP). The aspartate–glutamate carrier (AGC) is represented by $${J}_{\mathrm{AGC}}$$, a key step in calcium-dependent activation of glycolysis and the Krebs cycle. The latter two processes are both represented by $${J}_{\mathrm{PDH}}$$, the only non-voltage-dependent flux in the mitochondria model (Wacquier et al. 2016), described in Sect. 2.5.

### Calcium Handling: SR, Myoplasm, and Mitochondria

Calcium release from the SR via the ryanodine receptor (RyR) was modeled with both voltage and calcium dependence (Senneff and Lowery 2021):

4 $${J}_{\mathrm{RyR}}= \overline{{i }_{\mathrm{RyR}}}\times f\times \left(\sum_{i=1}^{4}{O}_{i}\right)\times \left({\left[{\mathrm{Ca}}^{2+}\right]}_{\mathrm{TSR}}-{\left[{\mathrm{Ca}}^{2+}\right]}_{\mathrm{TM}}\right)$$

where $\overline{{i }_{RyR}}$ is the maximal release rate, $f$ is a calcium-dependent inactivation gating variable, ${O}_{i\in \left\{1,\dots 4\right\}}$ are voltage-dependent activation gating variables, and ${\left[{\mathrm{Ca}}^{2+}\right]}_{\mathrm{TSR}}$ and ${\left[{\mathrm{Ca}}^{2+}\right]}_{\mathrm{TM}}$ are terminal SR and terminal myoplasmic calcium concentrations, respectively. Calcium enters the terminal myoplasmic space before diffusing into the bulk space, modulated by terminal and bulk mitochondrial calcium uptake via the MCU (Cortassa et al. 2003):

5 $${J}_{{\mathrm{MCU}}_{{\mathrm{M}}_{x}}}={V}_{\mathrm{MCU}}\frac{\frac{{\left[{\mathrm{Ca}}^{2+}\right]}_{x}}{{K}_{\mathrm{trans}}}{\left(1+ \frac{{\left[{\mathrm{Ca}}^{2+}\right]}_{x}}{{K}_{\mathrm{trans}}}\right)}^{3}\frac{2F\left(\Delta {\Psi }_{{\mathrm{M}}_{x}}-\Delta {\Psi }_{\mathrm{MCU}}^{\mathrm{o}}\right)}{\mathrm{RT}}}{{\left(1+\frac{{\left[{\mathrm{Ca}}^{2+}\right]}_{x}}{{K}_{\mathrm{trans}}}\right)}^{4}+\frac{L}{{\left(1+\frac{{\left[{\mathrm{Ca}}^{2+}\right]}_{x}}{{K}_{\mathrm{act}}}\right)}^{{n}_{a}}}\left(1-{e}^{\left\{-\frac{2F\left(\Delta {\Psi }_{{\mathrm{M}}_{x}}-\Delta {\Psi }_{\mathrm{MCU}}^{\mathrm{o}}\right)}{RT}\right\}}\right)}\quad x \epsilon \left\{\mathrm{TM}, M\right\}$$

The MCU is modeled to require relatively high concentrations of myoplasmic calcium to activate, capturing tight regulation of mitochondrial calcium observed in vivo. Maximal uptake rate, ${V}_{\mathrm{MCU}}$, was numerically tuned to elicit total calcium uptake per stimulus in the model matching data from skeletal muscle (Williams et al. 2013). ${K}_{\mathrm{trans}}$ is the ${K}_{\mathrm{d}}$ for translocated calcium, ${K}_{\mathrm{act}}$ is an activation constant, $L$ is the *K*_eq_ for conformational transitions, *n*_a_ is the activation cooperativity, and $\Delta {\Psi }_{\mathrm{MCU}}^{o}$ is the offset membrane potential.

Calcium extrusion from the mitochondria back into the myoplasm was modeled via the NCX (Cortassa et al. 2003):

6 $${J}_{{\mathrm{NCX}}_{{\mathrm{M}}_{x}}}={V}_{\mathrm{NCX}}\frac{{e}^{\left(\frac{0.5F(\Delta {\Psi }_{{\mathrm{M}}_{x}}-\Delta {\Psi }_{\mathrm{NCX}}^{o})}{RT}\right)}{\mathrm{e}}^{\mathrm{ln}\left(\frac{{\left[{\mathrm{Ca}}^{2+}\right]}_{\mathrm{x}}}{{\left[{\mathrm{Ca}}^{2+}\right]}_{{\mathrm{M}}_{\mathrm{x}}}}\right)}}{{\left(1+\frac{{K}_{\mathrm{Na}}}{{\left[{\mathrm{Na}}^{+}\right]}_{i}}\right)}^{3}\left(1+\frac{{K}_{\mathrm{Ca}}}{{\left[{\mathrm{Ca}}^{2+}\right]}_{{\mathrm{M}}_{x}}}\right)}\quad x \epsilon \{\mathrm{TM}, M\}$$

with maximal rate ${V}_{\mathrm{NCX}}$, offset membrane potential $\Delta {\Psi }_{\mathrm{NCX}}^{o}$, antiporter sodium constant ${K}_{\mathrm{Na}}$, and antiporter calcium constant ${K}_{\mathrm{Ca}}$. The intracellular sodium concentration, ${\left[{\mathrm{Na}}^{+}\right]}_{i}$, was held constant. In addition to ${J}_{\mathrm{MCU}}$ and ${J}_{\mathrm{NCX}}$, a bidirectional calcium flux was incorporated into the model representing the low-conductance state of the mitochondrial permeability transition pore (mPTP), originally formulated by Wacquier et al. (2016) and extended here to include transition to a high-conductance state:

7 $${J}_{{\mathrm{mPTP}}_{{\mathrm{M}}_{x}}}= \left\{\begin{array}{ll}{V}_{\mathrm{mPTP}}\left({\left[{\mathrm{Ca}}^{2+}\right]}_{x}-{\left[{\mathrm{Ca}}^{2+}\right]}_{{\mathrm{M}}_{x}}\right){e}^{{p}_{3}(\Delta {\Psi }_{{\mathrm{M}}_{x}})}& \mathrm{if} {\left[{\mathrm{Ca}}^{2+}\right]}_{{\mathrm{M}}_{x}}<{{C}_{t}}_{{\mathrm{M}}_{x}} \\ {k}_{t}\left[{V}_{\mathrm{mPTP}}\left({\left[{\mathrm{Ca}}^{2+}\right]}_{x}-{\left[{\mathrm{Ca}}^{2+}\right]}_{{\mathrm{M}}_{x}}\right){e}^{{p}_{3}(\Delta {\Psi }_{{\mathrm{M}}_{x}})}\right]& \mathrm{otherwise}\end{array}\quad x \epsilon \{\mathrm{TM}, M\}\right.$$

where ${C}_{\mathrm{t}}$ is the mitochondrial calcium threshold for low to high state transition, scaling the maximal flux rate, ${V}_{\mathrm{mPTP}}$, by factor ${k}_{\mathrm{t}}$. Voltage dependence of the mPTP is captured with an exponential function of the mitochondrial membrane potential multiplied by parameter ${p}_{3}$ (Wacquier et al. 2016).

Calcium uptake from the myoplasm back into the SR was modeled with an ATP-dependent SERCA pump (Senneff and Lowery 2021):

8 $${J}_{{\mathrm{SERCA}}_{x}}={\upsilon }_{\mathrm{SR}}\frac{{\left[{\mathrm{Ca}}^{2+}\right]}_{y}^{{n}_{S}}}{{K}_{\mathrm{SR}}+{\left[{\mathrm{Ca}}^{2+}\right]}_{y}^{{n}_{S}}}\left(\frac{{\left[\mathrm{ATP}\right]}_{y}}{{K}_{\mathrm{ATP}}+{\left[\mathrm{ATP}\right]}_{y}}\right)\quad x \epsilon \left\{\mathrm{TSR},\mathrm{ SR}\right\},\quad  y \epsilon \left\{\mathrm{TM}, M\right\}$$

where ${\upsilon }_{\mathrm{SR}}$ is the maximal pump rate, ${K}_{\mathrm{SR}}$ and ${K}_{\mathrm{ATP}}$ are Michaelis constants, and ${n}_{\mathrm{S}}$ is a Hill parameter.

Full model formulations for the concentration of calcium within each of the SR, myoplasm, and mitochondria sub-compartments can be found in Supplementary Material A.

### Calcium-Activated OXPHOS and ATP Dynamics

Activation of the ETC as a first step in OXPHOS requires a pool of available NADH to oxidize within the mitochondria. In the model, NADH begins to accumulate by first activating the AGC (Wacquier et al. 2016):

9 $${J}_{{\mathrm{AGC}}_{{\mathrm{M}}_{x}}}={V}_{\mathrm{AGC}}\left(\frac{{\left[{\mathrm{Ca}}^{2+}\right]}_{x}}{{K}_{\mathrm{AGC}}+{\left[{\mathrm{Ca}}^{2+}\right]}_{x}}\right)\left(\frac{{q}_{2}}{{q}_{2}+{\left[{\mathrm{Ca}}^{2+}\right]}_{{M}_{x}}}\right){e}^{{p}_{4}\Delta {\Psi }_{{\mathrm{M}}_{\mathrm{x}}}}\quad x \epsilon \{\mathrm{TM}, M\}$$

which contributes to redox equilibrium between glycolysis and ATP synthesis by transporting reducing agents from the myoplasm into the mitochondria (Amoedo et al. 2016). The AGC is activated by myoplasmic calcium, with maximal flux rate ${V}_{\mathrm{AGC}}$ and calcium dissociation constant ${K}_{\mathrm{AGC}}$, and additionally exhibits voltage dependence with coefficient ${p}_{4}$. Once the AGC flux rate begins to increase, the glycolytic pyruvate dehydrogenase (PDH) reaction and subsequent reduction of NAD+ to NADH as part of the Krebs cycle is initiated. This is captured in the model with ${J}_{\mathrm{PDH}}$ (Wacquier et al. 2016):

10 $${J}_{{\mathrm{PDH}}_{{M}_{\mathrm{x}}}}={V}_{\mathrm{GLY}}\left(\frac{1}{{q}_{1}+\frac{{\left[\mathrm{NADH}\right]}_{{\mathrm{M}}_{x}}}{{\left[{\mathrm{NAD}}^{+}\right]}_{{\mathrm{M}}_{x}}}}\right)\left(\frac{{\left[{\mathrm{Ca}}^{2+}\right]}_{{\mathrm{M}}_{x}}}{{q}_{2}+{\left[{\mathrm{Ca}}^{2+}\right]}_{{\mathrm{M}}_{x}}}\right)\quad x \epsilon \{\mathrm{TM}, M\} $$

where the first term represents NAD+ reduction, scaled by glycolytic rate ${V}_{\mathrm{GLY}}$, and ${q}_{1}$ is a Michaelis-like constant for NAD+ consumption. The second term represents mitochondrial calcium-dependent activation of the Krebs cycle, where ${q}_{2}$ is the half-activation constant. Once the Krebs cycle is activated in the model, the AGC is deactivated by mitochondrial calcium, according to the same threshold value ${q}_{2}$, as seen in the second term of Eq. (9).

The ETC is represented by a minimal respiration model that captures oxidation of NADH to extrude protons from the mitochondria (Bertram et al. 2006; Wacquier et al. 2016):

11 $${J}_{{\mathrm{ETC}}_{{\mathrm{M}}_{x}}}={V}_{\mathrm{ETC}}\left(\frac{{\left[\mathrm{NADH}\right]}_{{\mathrm{M}}_{x}}}{{q}_{3}+{\left[\mathrm{NADH}\right]}_{{\mathrm{M}}_{x}}}\right){\left(1+{e}^{\frac{\Delta {\Psi }_{{\mathrm{M}}_{x}}-{q}_{4}}{{q}_{5}}}\right)}^{-1} \quad x \epsilon \{\mathrm{TM}, M\}$$

where ${V}_{ETC}$ is the maximal rate of oxidation and proton extrusion, ${q}_{3}$ is a Michaelis constant for NADH consumption, and ${q}_{4}$ and ${q}_{5}$ are voltage dependence coefficients involved in proton extrusion. Downstream of the ETC, mitochondrial ATP synthesis by the F1F0 is initiated (Wacquier et al. 2016):

12 $${J}_{\mathrm{F}1\mathrm{F}{0}_{{M}_{\mathrm{x}}}}={V}_{\mathrm{F}1\mathrm{F}0}\left(\frac{{q}_{6}}{{q}_{6}+{\left[\mathrm{ATP}\right]}_{{\mathrm{M}}_{x}}}\right){\left(1+{e}^{\frac{{q}_{7}-\Delta {\Psi }_{{M}_{x}}}{{q}_{8}}}\right)}^{-1} \quad x \epsilon \{\mathrm{TM}, M\}$$

with maximal rate ${V}_{F1F0}$. The F1F0 is inhibited at high concentrations of mitochondrial ATP, according to threshold value ${q}_{6}$. Voltage dependence coefficients include ${q}_{7}$ and ${q}_{8}$. Translocation of mitochondrial ATP into the myoplasm via the ANT was modeled with dependence on mitochondrial and myoplasmic ADP/ATP ratios (Wu et al. 2007):

13 $${J}_{{\mathrm{ANT}}_{{\mathrm{M}}_{x}}}={V}_{\mathrm{ANT}}\left(\frac{{\left[\mathrm{ADP}\right]}_{x}}{{\left[\mathrm{ADP}\right]}_{x}+{\left[\mathrm{ATP}\right]}_{x}{e}^{-\theta F\Delta {\Psi }_{{\mathrm{M}}_{x}}/\mathrm{RT}}}-\frac{{\left[ADP\right]}_{{M}_{x}}}{{\left[\mathrm{ADP}\right]}_{{M}_{X}}+{\left[\mathrm{ATP}\right]}_{{\mathrm{M}}_{x}}{e}^{(1-\theta )F\Delta {\Psi }_{{\mathrm{M}}_{x}}/\mathrm{RT}}}\right)\left(\frac{1}{1+{k}_{\mathrm{m},\mathrm{ADP}}/{[\mathrm{ADP}]}_{x}}\right) \quad x \epsilon \{\mathrm{TM}, M\}$$

where ${V}_{\mathrm{ANT}}$ is the maximal rate of ATP transport, $\theta$ is an empirical parameter set from skeletal muscle, ${k}_{\mathrm{m},\mathrm{ADP}}$ is a Michaelis constant, $F$ is Faraday’s constant, $R$ is the gas constant, and $T$ is temperature.

Mitochondrial ATP and ADP concentrations were modeled with dependence on the balance between ATP synthesis and ATP translocation, scaled by sub-compartment volume:

14 $$\frac{\mathrm{d}{\left[\mathrm{ATP}\right]}_{{\mathrm{M}}_{x}}}{\mathrm{d}t}=\frac{1}{{V}_{{\mathrm{M}}_{x}}}\left({J}_{\mathrm{F}1\mathrm{F}{0}_{{\mathrm{M}}_{x}}}-{J}_{{\mathrm{ANT}}_{{\mathrm{M}}_{x}}}\right)\quad x \epsilon \{\mathrm{TM}, M\}$$

15 $$\frac{\mathrm{d}{\left[\mathrm{ADP}\right]}_{{\mathrm{M}}_{x}}}{\mathrm{d}t}=\frac{1}{{V}_{{\mathrm{M}}_{x}}}\left({J}_{{\mathrm{ANT}}_{{\mathrm{M}}_{x}}}-{J}_{\mathrm{F}1\mathrm{F}{0}_{{\mathrm{M}}_{x}}}\right)\quad x \epsilon \{\mathrm{TM}, M\}$$

a modification of the model by Wacquier et al. (2016) that utilized a conservation equation for adenine nucleotides. Similarly, a model for ADP accumulation in the myoplasm is presented in this study incorporating the amount of ATP consumed in the myoplasm per stimulus (${J}_{\mathrm{HYD}}$) and ADP diffusion between terminal and bulk myoplasm spaces:

16 $$\frac{\mathrm{d}{\left[\mathrm{ADP}\right]}_{x}}{\mathrm{d}t}=\frac{1}{{V}_{x}}\left[{J}_{{\mathrm{HYD}}_{x}}-{V}_{{M}_{x}}\left({J}_{\mathrm{AN}{{\mathrm{T}}_{\mathrm{M}}}_{x}}\right)-{\tau }_{\mathrm{ADP}}\left([\mathrm{AD}{\mathrm{P}]}_{\mathrm{TM}}-[\mathrm{AD}{\mathrm{P}]}_{\mathrm{M}}\right)\right]\quad x \epsilon \{\mathrm{TM}, M\}$$

where^1^${V}_{x}$ and ${V}_{{\mathrm{M}}_{x}}$ are sub-compartment volume parameters and ${\tau }_{\mathrm{ADP}}$ is a diffusion constant. The model of ATP consumption is described in Supplementary Material A, Sect. 5, along with the model description of ATP accumulation in the myoplasm.

### Whole Muscle Force Model and Incorporation of Calcium-Stimulated Apoptosis

The model of a single muscle fiber described in Sect. 2.1 was extended to a whole muscle comprised of 100 motor units to simulate the effect of reduced muscle mass, as a result of apoptosis, on force. To estimate individual motor unit forces, defined as ${F}_{i}$ for $i\in \{1,\dots ,100\}$, the single muscle fiber model was independently simulated 100 different times, wherein each simulation was initialized with a unique muscle fiber diameter.

Force output from each individual muscle fiber model was the result of an 8-stage crossbridge cycling model, described in detail in Senneff and Lowery (2021) and Shorten et al. (2007), which quantifies the concentration of attached myosin crossbridge formations performing force-generating power strokes. The resultant crossbridge concentration from each simulation, ${\left[{A}_{2}\right]}_{i}$ for $i\in \{1,\dots ,100\}$, was scaled by a constant value representing the number of muscle fibers innervated by each motor unit, defined as $n{o}_{fi{b}_{i}}$ for $i\in \{1,\dots ,100\}$. All motor unit forces were then summed to yield the whole muscle force, $Force$:

17 $$Force=\sum_{i=1}^{100}{F}_{i}=\sum_{i=1}^{100}{\left[{A}_{2}\right]}_{i}\times n{o}_{fi{b}_{i}} i \in \{1,\dots ,100\}$$

Fiber diameter for each independent model simulation ranged from 24.6 $\mathrm{\mu m}$ for the smallest motor unit ($i=1$) to 40.6 $\mathrm{\mu m}$ for the largest motor unit ($i=100$), and motor unit innervation number ranged from 21 fibers ($i=1$) to 841 fibers ($i=1$00). These parameter values originate from an earlier model developed to match motor unit action potential amplitudes observed experimentally in the first dorsal interosseous muscle (Botelho et al. 2019). An exponential function was used to interpolate the remaining parameter values:

18 $${p}_{i}={p}_{lb}+\frac{{e}^{\left(\frac{\mathrm{log}\left({p}_{ub}\left(100\right)-{p}_{lb}\left(100\right)\right)}{100}\times \left(i-1\right)\right)}}{100},\quad i \in \{1,\dots ,100\}$$

where $p$ is the parameter being interpolated, $lb$ is the lower bound value and $ub$ is the upper bound value.

To simulate the effect of OXPHOS inhibition on whole muscle force, a mathematical model of autophagy-apoptosis developed for mammalian cells (Tavassoly et al. 2015) was integrated into the model presented in this study and utilized to track the accumulation of apoptotic proteins during ECC. The model simulates cycling between autophagy and apoptosis by capturing a quantitative measure of internal stress within each fiber, ${S}$, which builds until it is relieved via autophagy, ${\left[Atphg\right]}$, the process of degradation of toxic proteins within muscle:

19 $$\frac{\mathrm{d}{S}_{i}}{\mathrm{d}t}=-\left({k}_{rb}+{k}_{ra}{\left[Atphg\right]}_{i}\right)\times {S}_{i}\quad i \in \{1,\dots ,100\}$$

where ${k}_{rb}$ and ${k}_{ra}$ are rate constants. The level of internal stress was computed for each of the 100 independent muscle fiber models, and then scaled by $n{o}_{fib}$, as done in the approach formulated in Eq. 17. As internal stress accumulates, the concentration of apoptotic caspase protein, $[CASP]$, increases in a calcium-dependent manner:

20 $$\frac{\mathrm{d}{\left[CASP\right]}_{i}}{\mathrm{d}t}={\gamma }_{\mathrm{C}}\left(Heav\left(\left[BH3\right]_{i}-{{\left[BCL2\right]}_{i}}_{{mit}}\right)-[CASP]_{i}\right) i \in \{1,\dots ,100\} $$

where ${\gamma }_{\mathrm{C}}$ is a rate constant and $\left[BH3\right]$ and ${\left[BCL2\right]}_{mit}$ are apoptotic proteins stimulated by the level of calcium within the bulk myoplasm. The concentration of active caspase protein transiently varied between 0 and 1 in the model as autophagy and apoptosis cycled (0 = autophagy, 1 = apoptosis).

Within the whole muscle model, an individual muscle fiber was determined to be ‘stuck’ in an apoptotic state if $[CASP]$ remained at 1 following repetitive tetanic stimulation of the muscle fiber for one second, instead of naturally returning to 0. If this was the case, the total force produced by the respective motor unit (i.e., the individual muscle fiber force scaled by $n{o}_{\mathrm{fib}}$) was subtracted from $Force$ in Eq. 17, emulating the effect of reduced muscle mass on whole muscle force in response to mitochondrial dysfunction.

### Simulation Details

The two-compartment model was implemented in Python 3.7.4 [MSC v.1915 64 bit (AMD64)] and simulated with IPython 7.8.0 in Spyder v. 3.3.6 from Anaconda. The model was numerically integrated using the odeint function from the SciPy package. This function solves systems of ordinary differential equations using the LSODA algorithm (Petzold 1983), an adaptive integrator capable of switching automatically between stiff and non-stiff solving, demonstrated to be more robust to integration failure compared to other solving methods for biological systems (Städter et al. 2021).

## Results

### Muscle Fiber Force Generation

**Fig. 2:**
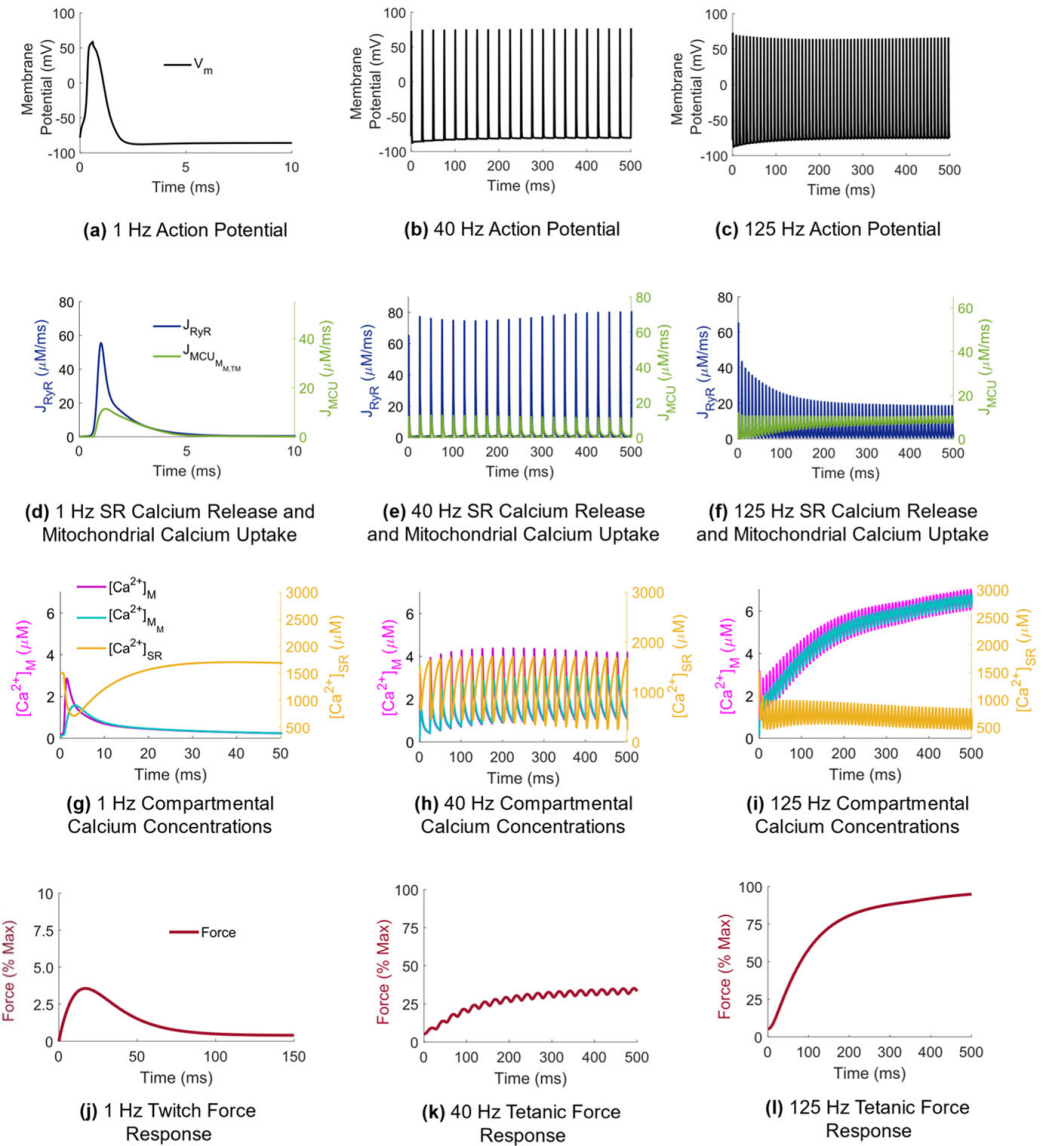
(Color figure online) Force generation in response to increased stimulation frequency. **a**–**c** Action potential generation along the sarcolemma (${V}_{\mathrm{m}}$). **d**–**f** Calcium release flux out of the SR (${J}_{\mathrm{RyR}}$) and calcium uptake into the mitochondria in both the terminal and bulk spaces (${J}_{{\mathrm{MCU}}_{{M}_{\mathrm{M},\mathrm{TM}}}}$). **g**–**i** Compartmental calcium concentrations within the bulk myoplasm (${\left[{\mathrm{Ca}}^{2+}\right]}_{\mathrm{M}}$), bulk mitochondria (${\left[{\mathrm{Ca}}^{2+}\right]}_{{M}_{\mathrm{M}}}$), and bulk SR (${\left[{\mathrm{Ca}}^{2+}\right]}_{\mathrm{SR}}$). **j**–**l** Force generated, presented as the percentage of maximal force elicited at 125 Hz stimulation frequency

Following action potential generation, mitochondrial calcium uptake occurred after a short delay once calcium was released from the SR (Fig. 2d).

The rate of mitochondrial calcium uptake remained transiently high away from its initial value as mitochondria began to accumulate more calcium during tetanic stimulation at 40 and 125 Hz (Fig. 2e, f), contributing to depletion of SR calcium stores (Fig. 2h, i).

The force twitch duration was approximately 150 ms and peaked at 4% maximal force (Fig. 2j). In response to 40 Hz stimulation, force tetanized at approximately 40% maximal force (Fig. 2k). During a single force twitch, most of the calcium released from the SR remained in the myoplasm, with mitochondrial calcium peaking after a delay of 3 ms to approximately 50% of the myoplasmic value (Fig. 2g). However, the ratio of peak mitochondrial calcium concentration to peak myoplasmic calcium concentration increased with increasing stimulation frequency, from 0.75 at 40 Hz to 0.95 at 125 Hz stimulation (Fig. 2h, i).

### Calcium-Activated OXPHOS During Tetanic Contractions

**Fig. 3:**
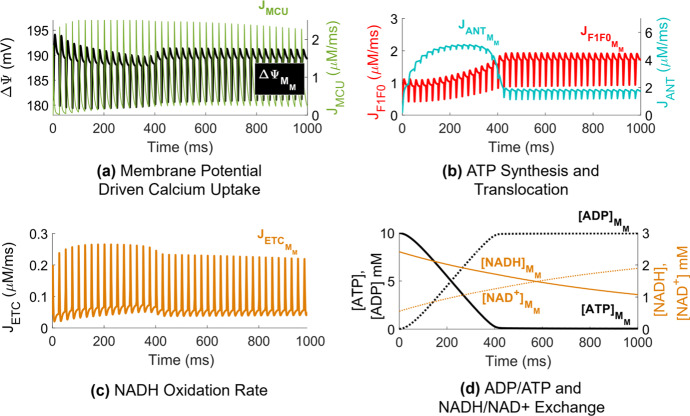
(Color figure online) Calcium-activated OXPHOS during 40 Hz stimulation. **a** Mitochondrial membrane potential in the bulk mitochondria space ($\Delta {\Psi }_{{M}_{\mathrm{M}}}$) and total mitochondrial calcium uptake averaged across both the terminal and bulk mitochondria (${J}_{\mathrm{MCU}}$). **b** Rate of mitochondrial ATP synthesis in the bulk mitochondria (${J}_{F1F{0}_{{M}_{\mathrm{M}}}}$) and rate of translocation of ATP from the bulk mitochondria into the bulk myoplasm (${J}_{{\mathrm{ANT}}_{{M}_{\mathrm{M}}}}$). **c** Rate of NADH oxidation by the ETC in the bulk mitochondria (${J}_{{\mathrm{ETC}}_{{M}_{\mathrm{M}}}}$). **d** Concentration of mitochondrial ADP (${\left[\mathrm{ADP}\right]}_{{M}_{\mathrm{M}}}$) and ATP (${\left[\mathrm{ATP}\right]}_{{M}_{\mathrm{M}}}$) presented against the concentration of mitochondrial NADH (${\left[\mathrm{NADH}\right]}_{{M}_{\mathrm{M}}}$) and NAD + (${\left[{\mathrm{NAD}}^{+}\right]}_{{M}_{\mathrm{M}}}$) in the bulk mitochondria

Prolonged mitochondrial calcium uptake during repetitive stimulation at 40 Hz resulted in temporary depolarization of $\Delta \Psi$ below its resting value (Fig. 3a).

Accumulation of mitochondrial calcium during this period increased the supply of NAD+ ions following Krebs cycle stimulation (Fig. 3d), activating the flux through the ETC (Fig. 3c). ATP was rapidly synthesized by the F1F0 to meet the energetic demand for force generation during the onset of tetanic stimulation (Fig. 3b). This coincided with a decrease in mitochondrial ATP (Fig. 3d) as the ANT translocated ATP into the myoplasm at an elevated rate (Fig. 3b). Once the mitochondrial ADP/ATP ratio increased (Fig. 3d), the flux through the F1F0 saturated (Fig. 3b) coinciding with a plateau of the force response (Fig. 2k).

### Mitochondrial Dysfunction

#### Effect on Myoplasmic ADP and ATP

**Fig. 4:**
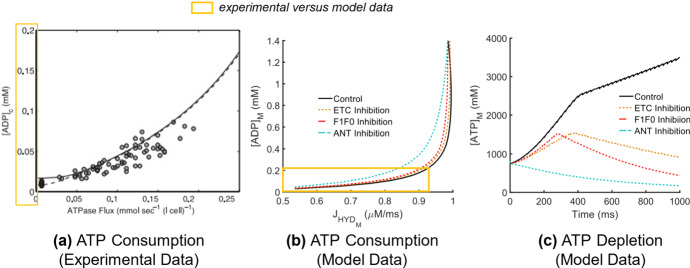
(Color figure online) Reduced myoplasmic ATP in response to OXPHOS inhibition. **a** Experimental myoplasmic ADP concentration versus rate of ATP consumption, reproduced with permission from Wu et al. (2007). **b** ATP consumed in the bulk myoplasm (${J}_{{\mathrm{HYD}}_{\mathrm{M}}}$) versus ADP concentration (${\left[\mathrm{ADP}\right]}_{\mathrm{M}}$). **c** Reduced ATP in the bulk myoplasm (${\left[\mathrm{ATP}\right]}_{\mathrm{M}}$) during repetitive stimulation in response to OXPHOS impairment

The relationship between the rate of ATP consumption and ADP concentration within the myoplasm in the model was softly exponential at rest (Fig. 4b), consistent with experimental data collected from human skeletal muscle during steady-state exercise (Fig. 4a). To reach a steady-state force output in the model in response to continuous tetanic stimulation, the amount of ATP in the myoplasm increased steeply during the transient phase (force generation phase, approx. 0–350 ms) until force level was maintained, slowing the rate of ATP accumulation (Fig. 4c, Control, Fig. 2k).

ANT inhibition led to the greatest increase in ATP demand compared to F1F0 and ETC inhibition (Fig. 4b), coinciding with the lowest availability of myoplasmic ATP (Fig. 4c). Furthermore, in the case of ANT inhibition, there was no initial increase in myoplasmic ATP present at the onset of muscle fiber stimulation during the transient force phase (Fig. 3b), indicating that ATP is accumulating within the mitochondria due to ANT block. This was less severe in the case of ETC and F1F0 inhibition, where ATP levels did marginally increase during force onset, but to lesser values than in the control case (Fig. 4c). As steady-state force was reached in the model, ATP levels began to drop in response to OXPHOS impairments, suggesting that ATP levels are more difficult to maintain in the model to hold a steady-state force, particularly in the case of F1F0 inhibition, exhibiting the steepest decline. To explore the mechanisms underlying ATP decline in the model further, changes in $\Delta \Psi$ and mitochondrial calcium handling were explored next.

#### Effect on $\Delta \boldsymbol{\Psi }$, Calcium Handling, and Compartmental Calcium

**Fig. 5:**
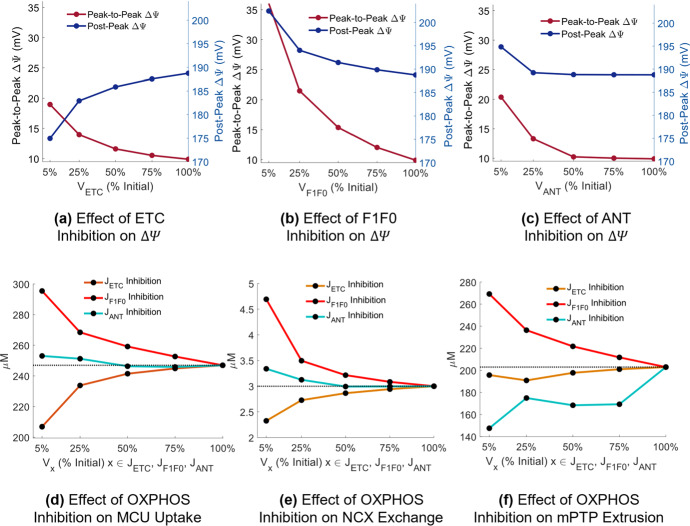
(Color figure online) Effect of OXPHOS impairment on the mitochondrial membrane potential and mitochondrial calcium handling. Inhibition of **a** ETC (via progressively reducing ${V}_{\mathrm{ETC}}$), **b** F1F0 (via progressively reducing ${V}_{F1F0}$) and **c** ANT (via progressively reducing ${V}_{\mathrm{ANT}}$). **d** Net amount of calcium taken up into the mitochondria by the MCU per stimulus. **e** Net amount of calcium extruded into the myoplasm by the NCX per stimulus. **f** Net amount of calcium extruded into the myoplasm by the mPTP per stimulus. Subplots **d**–**f** presented in response to either reduced ${V}_{\mathrm{ETC}}$, ${V}_{F1F0}$, or ${V}_{\mathrm{ANT}}$. Subplots **a**–**f** *x *axis corresponds to the flux rate as a percentage of its initial value, i.e., 5% refers to 95% inhibition

Progressive inhibition of the ETC, F1F0, and ANT was performed in steps of 25%^2^ to observe the effect of each impairment individually on $\Delta \Psi$ and mitochondrial calcium handling (Fig. 5).

Deviation of $\Delta \Psi$ from its initial value of 190 mV at rest to its peak value following stimulation was quantified, labeled ‘Peak-to-Peak $\Delta \Psi$’ in Fig. 5a–c. The resting value of $\Delta \Psi$ post-stimulation was also quantified, labeled ‘Post-Peak $\Delta \Psi$’ in Fig. 5a–c. In the case of near-maximal OXPHOS inhibition (5% of the initial flux rate), Peak-to-Peak $\Delta \Psi$ increased from a 10 mV difference to a 20 mV difference in response to ETC inhibition (Fig. 5a), from a 10 mV to a 35 mV difference following F1F0 inhibition (Fig. 5b), and from a 10 mV to a 20 mV difference following ANT inhibition (Fig. 5c), demonstrating a loss of controlled regulation of $\Delta \Psi$ for all three cases in the model. ETC inhibition resulted in depolarization of Post-Peak $\Delta \Psi$ from 190 to 175 mV (Fig. 5a), but both F1F0 and ANT inhibition resulted in hyperpolarization of Post-Peak $\Delta \Psi$, to 202 mV (Fig. 5b) and to 195 mV (Fig. 5c), respectively.

**Fig. 6:**
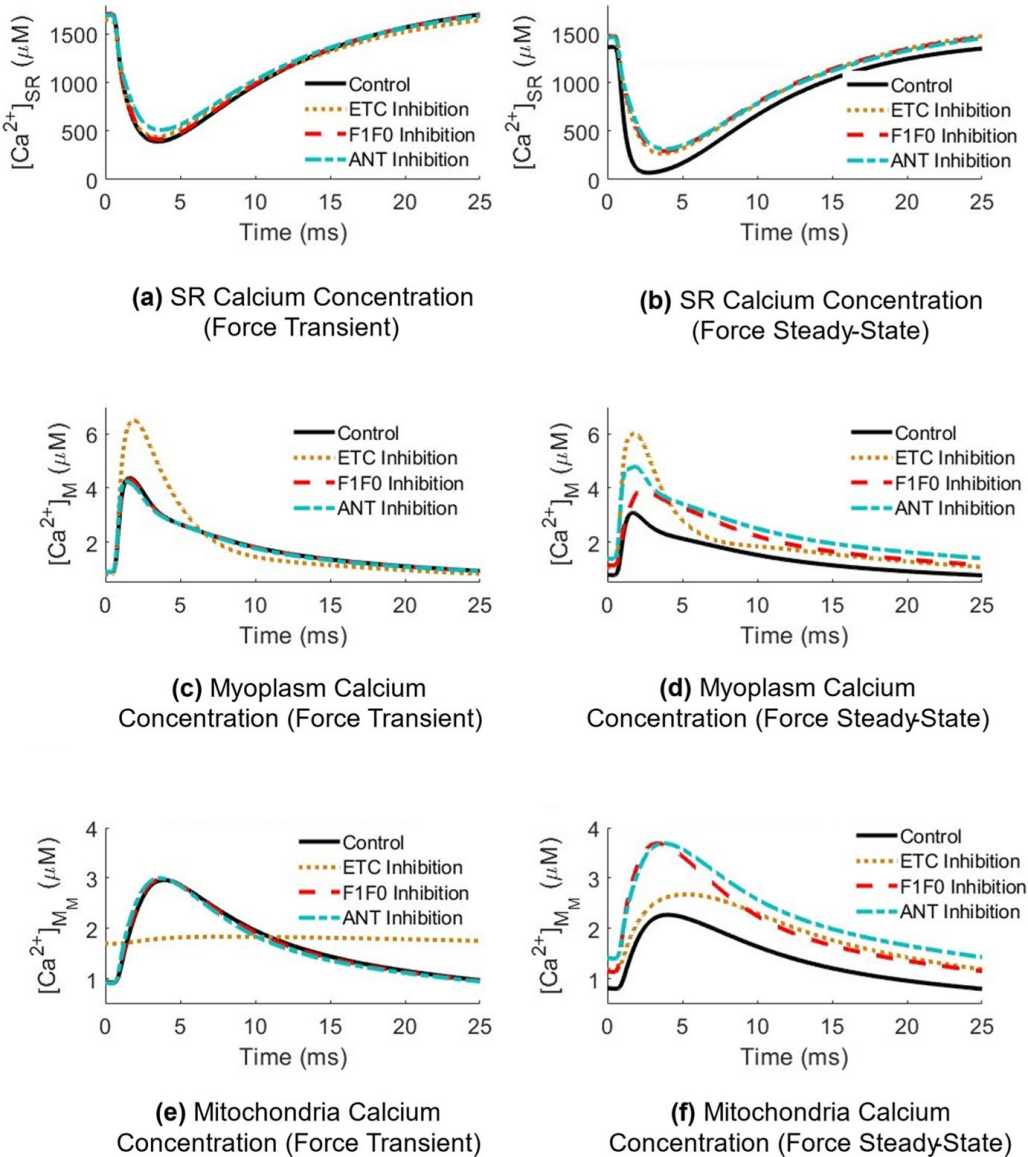
(Color figure online) Effect of OXPHOS inhibition on bulk compartmental calcium concentrations during distinct force phases in response to continuous stimulation at 40 Hz. The *force transient* phase is defined during the time period when force is increasing, preceding tetanization (*force steady-state* phase). Results are taken as a snapshot of activity in response to an action potential train and normalized to a zero-time *x *axis. **a**, **b** Calcium concentration within the bulk SR (${\left[{\mathrm{Ca}}^{2+}\right]}_{\mathrm{SR}}$) in response to OXPHOS inhibition during a single action potential within the transient (left) or steady-state (right) contraction portion. **c**, **d** Calcium concentration within the bulk myoplasm (${\left[{\mathrm{Ca}}^{2+}\right]}_{\mathrm{M}}$). **e**, **f** Calcium concentration within the bulk mitochondria (${\left[{\mathrm{Ca}}^{2+}\right]}_{{M}_{\mathrm{M}}}$.)

Depolarization of $\Delta \Psi$ in response to ETC inhibition coincided with reduced mitochondrial calcium uptake (Fig. 5d), leading to elevated myoplasmic calcium compared to the control case during both the transient (Fig. 6c) and steady-state (Fig. 6d) force generation phases. Contrastingly, F1F0 and ANT inhibition resulted in elevated myoplasmic calcium during steady-state force, but to a lesser degree than ETC inhibition in the model, suggesting there was little effect of $\Delta \Psi$ hyperpolarization on myoplasmic calcium handling during the onset of force generation. Hyperpolarization did coincide with increased MCU and NCX activity (Fig. 5d, e); however, the net amount of calcium moved from the mitochondria into the myoplasm via the mPTP increased in response to F1F0 inhibition but decreased in response to ANT inhibition (Fig. 5f), suggesting that disruptions in mitochondrial calcium handling are not the primary driver of myoplasmic calcium accumulation in the case of ANT inhibition.

**Fig. 7:**
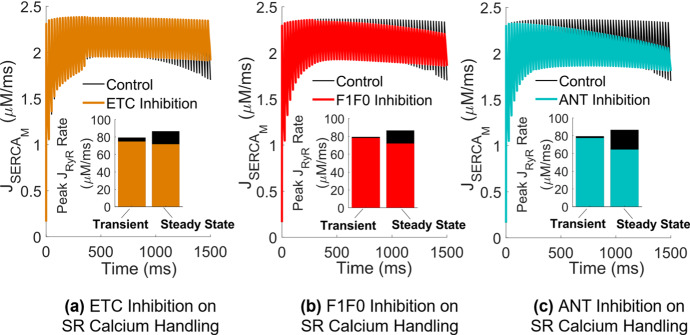
(Color figure online) Effect of OXPHOS inhibition on myoplasmic calcium handling during distinct force phases. **a** Rate of calcium uptake via the SERCA pump in response to ETC inhibition, **b** in response to F1F0 inhibition, and **c** in response to ANT inhibition during sustained contractions at 40 Hz. Subplots **a**–**c** the peak rate of SR calcium release via the RyR during the force transient and force steady-state contraction portions

Disruptions in mitochondrial calcium handling (Fig. 5) combined with reduced myoplasmic ATP levels (Fig. 4) led to a 20% reduction in the SERCA pump rate during the steady-state force phase in the ANT inhibited model compared to the control (Fig. 7c).

This contributed to higher peak myoplasmic calcium levels relative to the F1F0 inhibited model (Fig. 6d), where there was only an 8% pump rate reduction (Fig. 7b). SERCA pump rate was the least affected by ETC inhibition (Fig. 7a). ANT inhibition in the model presented with the greatest reduction in SR calcium release compared to the F1F0 and ETC inhibited models during both the transient and steady-state portions of force generation (Fig. 7c).

#### Effect of Calcium-Stimulated Apoptosis on Whole Muscle Force

**Fig. 8:**
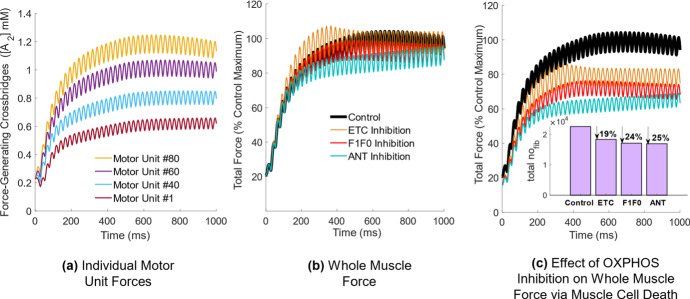
(Color figure online) Whole muscle force generation in the control model and in response to OXPHOS inhibition during continuous stimulation at 40 Hz. **a** Individual motor unit force generation for four representative motor units of increasing size. **b** Whole muscle force in the model and in response to OXPHOS inhibition unadjusted for calcium-stimulated apoptosis and **c** adjusted for calcium-stimulated apoptosis to quantify the reduction in muscle mass

To examine the effect of prolonged elevated myoplasmic calcium on muscle force, the model was extended from a single fiber to a whole muscle comprised of 100 motor units of increasing fiber innervation number. Individual motor unit forces increased with increasing fiber diameter (Fig. 8a), and all 100 individual motor unit forces were summed to yield the total control force in Fig. 8a, b.

When comparing total control force to each impairment case, this was done in the scenario where muscle fibers were not removed from the total force calculation (NO accounting for calcium-stimulated apoptosis) presented in Fig. 8b, versus the scenario where entire motor unit forces WERE removed from the total force calcium (accounting for calcium-stimulated apoptosis), presented in Fig. 8c. In the first scenario, ETC inhibition demonstrated no effect on steady-state force level, while F1F0 and ANT inhibition led to a drop in steady-state force (Fig. 8b). In fact, there was an increase in force in response to ETC inhibition during the force transient phase. In the second scenario, whole muscle force dropped for all three cases during both force onset and steady-state force, corresponding to multiple muscle fibers being ‘stuck’ in an apoptotic state in the model due to myoplasmic calcium accumulation (Fig. 8c). Quantifying this further, approximately 19% of muscle fiber forces were lost in response to ETC inhibition, 24% in response to F1F0 inhibition, and 25% in response to ANT inhibition in the model (Fig. 8c). Maximal steady-state force decreased by approximately 20% in the ETC inhibited model, 28% in the F1F0 inhibited model and 36% in the ANT inhibited model (Fig. 8c).

## Discussion

An integrated model of ECC and calcium-activated OXPHOS was developed to simulate the regulatory role of mitochondria on calcium homeostasis and the consequences of mitochondrial dysfunction during sustained contractions. The model represents a first step in incorporating the effect of calcium-activated OXPHOS in skeletal muscle contraction models. Mitochondrial dysfunction was introduced via three distinct OXPHOS impairments of either the ETC, F1F0, or ANT to independently examine their effect on myoplasmic calcium and whole muscle force.

### Calcium Handling, OXPHOS, and Force Generation

Mitochondrial calcium levels peaked at approximately 50% of the myoplasmic calcium concentration during a single twitch (Fig. 2g). This increased to 75% during 40 Hz stimulation and to 95% during 125 Hz stimulation (Fig. 2h, i), demonstrating the protective role of mitochondria on myoplasmic calcium levels during sustained contractions, also observed in vivo in mice (Rudolf et al. 2004; Rossi et al. 2011). Mitochondrial calcium uptake was tightly coupled to $\Delta \Psi$ in the model. At the onset of force generation basal MCU rate increased, coinciding with depolarization of $\Delta \Psi$ from its initial resting value, that was maintained between stimuli (Fig. 3a). This transient resting potential offset has been reported experimentally in isolated skeletal muscle fibers (Casas et al. 2010; Mammucari et al. 2015; Díaz-Vegas et al. 2018). Once a steady-state force was reached in the model, however, $\Delta \Psi$ returned to its physiological resting value between action potentials within a train, consistent with in vivo observations in mice demonstrating that $\Delta \Psi$ is maintained during repetitive stimulation (Rossi et al. 2011).

Increased myoplasmic and mitochondrial calcium levels following muscle excitation stimulated NADH production in the model (Fig. 3d) via activation of the AGC and subsequent induction of the glycolytic PDH reaction (Eqs. 9,10). Accumulation of NADH drove the flux through the ETC (Fig. 3c), causing a transient increase in the rate of ATP synthesis (Fig. 3b). This transient rate increase occurs during force onset, yielding a high mitochondrial ATP/ADP ratio (Fig. 3d), eliciting ATP transport into the myoplasm via the ANT (Fig. 3b). ATP was steadily consumed in the myoplasm during each contraction until the force tetanized, coinciding with a rise in myoplasmic ADP (Fig. 4b, Control) as ATP synthesis rate slowed (Figs. 3b, 4c, Control). These transient-to-steady-state changes in the mitochondrial ATP/ADP ratio driving OXPHOS state transitions are consistent with earlier simulation studies emulating experimental observations in isolated muscle mitochondria under both low and high ADP conditions (Korzeniewski and Froncisz 1991).

### Effect of Mitochondrial Dysfunction

Accumulation of myoplasmic calcium occurred in the model through different primary mechanisms for each OXPHOS impairment. ETC inhibition decreased the amount of calcium taken up into the mitochondria via the MCU per stimulus (Fig. 5d), resulting in elevation of myoplasmic calcium and increased force output compared to the control case during the transient force phase (Fig. 6c), suggesting that ETC impairment may have a greater effect on force development than on force maintenance during voluntary contraction. Conversely, F1F0 and ANT inhibition had no effect on myoplasmic calcium during force onset (Fig. 6c). Additionally, myoplasmic calcium levels were less elevated in the case of F1F0 and ANT inhibition compared to ETC inhibition during the force steady-state phase (Fig. 6d), suggesting that although all three cases present with $\Delta \Psi$ dysregulation (Fig. 5), depolarization of $\Delta \Psi$ may have a greater impact on calcium homeostasis in skeletal muscle than hyperpolarization of $\Delta \Psi$.

ETC impairments in skeletal muscle have been observed in Parkinson’s disease patients; however, the nature of these abnormalities are still debated (Winkler-Stuck et al. 2005; Kelly et al. 2014). Experiments performed in Parkinsonian mice with a *DJ-1* genetic knockout found that muscle fibers presented with increased resting calcium and reduced calcium release from the SR into the myoplasm, which was attributed to a loss of myoplasmic ATP, as no change in muscle fiber excitability was observed (Shtifman et al. 2011). A similar relationship was seen in the model, wherein resting calcium levels were elevated (Fig. 6d) and calcium release rates were reduced (Fig. 7a–c), alongside myoplasmic ATP depletion (Fig. 4c), with no change in the resting membrane potential across the sarcolemma (not shown). This occurred in all OXPHOS impairment cases, but to varying degrees with each feature.

ANT inhibition presented with the highest demand for ATP during force generation (Fig. 4b), consistent with skeletal muscle data gathered in ANT-deficient and ETC-deficient humans during steady-state exercise (Wu et al. 2007). This coincided with the largest reduction in steady-state whole muscle force in response to calcium-stimulated apoptosis (Fig. 8c). Elevated myoplasmic calcium in the case of ANT inhibition was driven primarily by reduced calcium uptake into the SR via the SERCA pump (Fig. 7c), which worsened over time as more ATP was consumed in the myoplasm without being replenished by mitochondrial sources (Fig. 4c), leaving less available for SERCA function. SERCA impairments have been demonstrated to result in a slowed decline of myoplasmic calcium (Westerblad and Allen 1994), similarly observed in the model (Fig. 6d). Additionally, ANT inhibition presented with the greatest build-up of mitochondrial calcium (Fig. 6f) due to the largest reduction in the net amount of calcium extruded via the mPTP per stimulus (Fig. 5f). Conversely, the greatest increase in the net amount of calcium extruded via the mPTP occurred in response to F1F0 inhibition (Fig. 5f), despite both ANT and F1F0 impairments hyperpolarizing $\Delta \Psi$. This contrasting result suggests that disruptions in mitochondrial calcium handling are not the primary driver of myoplasmic calcium accumulation in the case of ANT inhibition, but instead the loss of ATP.

When considering the role of F1F0 inhibition on force loss, experimental work in mouse muscle has suggested apoptosis may result from a combination of factors including $\Delta \Psi$ hyperpolarization and mPTP opening (a precursor to apoptosis) (Briston et al. 2017), as well as altered BCL2 activity (Ciammola et al. 2006), a component of the calcium-stimulated apoptosis model described in Sect. 2.6, Eq. 20 but not explored in the current study. Increased mPTP conductance as a result of $\Delta \Psi$ hyperpolarization was exacerbated by impaired SERCA activity as a consequence of low myoplasmic ATP (Fig. 4c) to induce elevated myoplasmic calcium. Additionally, the rate of ATP depletion in the model was highest in the F1F0 inhibition case (Fig. 4c), suggesting more difficulty in maintaining steady-state force in the model compared to ETC and ANT impaired muscle (Fig. 8b, c). This could be explored further in the context of Huntington’s disease patients, whom have similarly presented with lower levels of muscle ATP content in response to reduced ATP synthesis via the F1F0 (Lodi et al. 2000).

### Model Limitations

While this model is the first in skeletal muscle to integrate mitochondrial calcium handling and calcium-activated OXPHOS with a physiological model of ECC, several important model aspects were simplified. Critically, the mitochondria model used in this study was based primarily on a model in HeLa cells (Wacquier et al. 2016). However, this model was derived from first principles in computational modeling of mitochondria originally developed for pancreatic beta cells (Magnus and Keizer 1998a, b) and reduced into a minimal model (Bertram et al. 2006). The minimal model uses a simplified mathematical representation of the glycolytic pathway and the Krebs cycle, as well as combines each electron transport chain complex into a single mathematical expression for NADH oxidation and proton extrusion rates (Wacquier et al. 2016). For this study, these model assumptions are conserved as the focus is on calcium-driven activation of mitochondrial dynamics versus the inner workings of the glycolytic pathway or electron transfer.

Calcium uptake and exchange relationships within the mitochondria are mathematically formulated based on cardiac muscle (Cortassa et al. 2003), adapted from work by Magnus and Keizer (1998a, b), except for the mPTP model which was extended from Wacquier et al. (2016). The latter model incorporated a calcium-dependent transition from a low to a high conductance state indicative of apoptotic induction; however, it is noted here that conductance state transitions are not strictly calcium-dependent, as modeled in Eq. 7, but are also dependent on $\Delta \Psi$ (Wacquier et al. 2020)*.* Lack of $\Delta \Psi$ dependence on conductance transitioning is considered a limitation in this study that may have underrepresented the impact of altered mPTP function in the model.

ATP/ADP exchange between the mitochondria and myoplasm was modeled as described for skeletal muscle (Wu et al. 2007); however, the inner and outer mitochondrial membranes were lumped in this study. It is additionally noted here that mitochondrial creatine kinase, which has been demonstrated to regulate mitochondrial ATP levels, was not incorporated into the mitochondria model. This is particularly important to consider in the case of F1F0 inhibition, as creatine kinase has been implicated as a protector against mPTP induction (Schlattner et al. 2006). Furthermore, altered levels of creatine kinase have been linked to impaired calcium homeostasis and dysfunctional ROS clearance during heart failure (Keceli et al. 2022), which could be explored with a modified version of the model presented here for cardiac muscle.

Mitochondria model parameters in this study were adapted for muscle where possible using existing computational studies in both skeletal (Korzeniewski and Zoladz 2001; Wu et al. 2007) and cardiac muscle (Cortassa et al. 2003). The lack of experimental data to describe skeletal muscle mitochondria presents challenges for model validation, with existing observations varying between in vitro and in vivo studies under different energy demands. To address this, the model was tuned to capture the relative contribution of mitochondrial calcium uptake to myoplasmic calcium levels reported experimentally in skeletal muscle (Rudolf et al. 2004; Yi et al. 2011; Williams et al. 2013). Further iterations of the model will enable closer alignment with skeletal muscle mitochondrial function. Despite these limitations, the model captured the fundamental relationship between calcium and stimulation of mitochondrial ATP synthesis observed experimentally (Glancy and Balaban 2012; Glancy et al. 2013).

The role of muscle fiber type dependence was not considered in this study. When extending the single fiber model to many fibers and motor units comprising a whole muscle, only fiber diameter was varied across simulations, while all other model parameters were held constant. Muscle fiber force loss as a result of calcium-stimulated apoptosis in this model was more prominent in larger diameter fibers, representative of fast twitch fiber types. This was due to greater SR calcium release per stimulus as a result of higher tubular radial conductivity in the model, increasing the sensitivity of larger fibers to calcium overload. The relationship between fiber type and calcium-stimulated apoptosis could be explored further by extending not just the slow twitch fiber type model from Senneff and Lowery (2021) to incorporate mitochondria, as is done in the model presented in this study, but the fast twitch fiber type model as well.

Interestingly, experimental work in Amyotrophic lateral sclerosis mouse models presenting with mutations in the *SOD1* gene, involved in ROS clearance, have demonstrated that there was a selective loss of fast twitch skeletal muscle fibers versus slow twitch skeletal muscle fibers (Milani et al. 2013). This was suggested to be due to less expression of *Nrf2*, contributing to reductions in cellular damage associated with tissue injury. The effects of ROS accumulation on myoplasmic calcium handling were not explored in this study at present.

## Conclusion

A model of calcium-activated OXPHOS was integrated into a model of ECC in skeletal muscle for the first time. The model captures experimentally observed accumulation of mitochondrial calcium during sustained contractions and resulting activation of ATP synthesis via OXPHOS to meet the muscle energy demand for force generation. Progressive OXPHOS inhibition resulted in altered $\Delta \Psi$ and pathological myoplasmic calcium accumulation due to reduced mitochondrial calcium uptake in response to ETC inhibition, increased conductance of the mPTP combined with a loss of myoplasmic ATP in response to F1F0 inhibition, and impaired SERCA pump activity as a result of myoplasmic ATP depletion in response to ANT inhibition. OXPHOS impairments ultimately led to whole muscle force loss as a result of calcium-stimulated muscle fiber apoptosis. The interactions between $\Delta \Psi$, mitochondrial calcium handling, and OXPHOS are complex; however, the model offers an exploratory tool to achieve insights into changes occurring during ECC associated with disrupted calcium homeostasis and muscle weakness in neurodegenerative and muscular diseases, as well as an overall understanding of mitochondrial function in skeletal muscle.

## Supplementary Information

Below is the link to the electronic supplementary material.

Supplementary file1 (PDF 172 KB)

Supplementary file2 (PDF 105 KB)

## Footnotes

1 The ‘$-{\tau }_{\mathrm{ADP}}\left({\mathrm{ADP}}_{\mathrm{TM}}-{\mathrm{ADP}}_{\mathrm{M}}\right)$’ term becomes $+{\tau }_{\mathrm{ADP}}({\mathrm{ADP}}_{\mathrm{TM}}-{\mathrm{ADP}}_{\mathrm{M}}$) when $x=M$.

2 Maximal inhibition of each flux rate was considered to be 5% of the initial flux rate under control conditions, instead of 0%, in order to avoid complete $\Delta \Psi$ collapse in the model.
