# Supplementary material for: Computational Model of the Effect of Mitochondrial Dysfunction on Excitation–Contraction Coupling in Skeletal Muscle
Source: Bull Math Biol. 2022 Sep 17;84(11):123. doi: 10.1007/s11538-022-01079-3 (PMC9482608; doi:10.1007/s11538-022-01079-3)
Supplement: Supplementary file 1 — Supplementary file1 (PDF 172 KB) [file 11538_2022_1079_MOESM1_ESM.pdf]

Supplementary Material A

Note: This supplementary material introduces model equations for integration of the model of excitation-contraction coupling with a model of calcium-activated OXPHOS that were not described in the main text. Equations that refer strictly to ECC components that were not modified in this study can be found in more detail in Senneff and Lowery (2021). This includes the model of muscle excitation, and all ionic channels and pumps associated, to govern sarcolemma and transverse-tubular system action potentials. This also includes tracking of the terminal and bulk SR calcium concentration, as well as all reactions between calcium ions and calsequestrin, and calcium ions and phosphate within the SR. Descriptions of all reactions between calcium ions within the myoplasm and buffers magnesium, parvalbumin, and ATP can be found, as well as a referenced description of a multi-state crossbridge cycling model to generate force, initiated by calcium-troponin binding.

1. Terminal and Bulk Myoplasm Calcium Concentrations

Calcium concentration changes within both the terminal and bulk myoplasmic space were updated in this study to incorporate the regulation of mitochondrial calcium handling on myoplasmic calcium levels. The rate of change of calcium within the terminal myoplasm was modeled as:

$$\begin{aligned}\frac{d[Ca^{2+}]_{TM}}{dt} = \frac{1}{V_{TM}} \Big[\Big(-J_{MCU_{M_{TM}}} + J_{NCX_{M_{TM}}} - J_{mPTP_{M_{TM}}} \Big) + I_{CaT} + J_{RyR} \\ - J_{SERCA_{TSR}} + L_e([Ca^{2+}]_{TSR} - [Ca^{2+}]_{TM}) \\ - t_R([Ca^{2+}]_{TM} - [Ca^{2+}]_M) \Big] \\ - \big(k_{CATP}^{on}[Ca^{2+}]_{TM}[ATP]_{TM} - k_{CATP}^{off}[CaATP]_{TM} \big) \\ - \big(k_{Parv}^{on}[Ca^{2+}]_{TM}(Parv_{tot} - [CaParv]_{TM} - [MgParv]_{TM}) \\ - k_{Parv}^{off}[CaParv]_{TM} \big)\end{aligned} \quad (1.1)$$

and in the bulk myoplasm was modeled as:

$$
\begin{aligned}
\frac{d[Ca^{2+}]_M}{dt} = \frac{1}{V_M}\Big[&\Big(-J_{MCU_{M_M}} + J_{NCX_{M_M}} - J_{mPTP_{M_M}}\Big) - J_{SERCA_{SR}} + L_e([Ca^{2+}]_{SR} \\
&- [Ca^{2+}]_M) + t_R([Ca^{2+}]_{TM} - [Ca^{2+}]_M)\Big] \\
&- \big(k^{on}_{CATP}[Ca^{2+}]_M[ATP]_M - k^{off}_{CATP}[CaATP]_M\big) \\
&- (k^{on}_{Parv}[Ca^{2+}]_M(Parv_{tot} - [CaParv]_M - [MgParv]_M) \\
&- k^{off}_{Parv}[CaParv]_M) - (k^{on}_T[Ca^{2+}]_M[T_0] - k^{off}_T[CaT] \\
&+ k^{on}_T[Ca^{2+}]_M[CaT] - k^{off}_T[CaCaT] + k^{on}_T[Ca^{2+}]_M[D_0] \\
&- k^{off}_T[D_1] + k^{on}_T[Ca^{2+}]_M[D_1] - k^{off}_T[D_2])
\end{aligned}
\tag{1.2}
$$

where V_{TM} and V_M represent the volume of the terminal and bulk myoplasmic spaces, respectively. I_{CaT} is a transverse-tubule calcium channel described in Eqn. 7 in Senneff and Lowery (2021). This channel is modeled with both voltage and calcium-dependence. Calcium release from the sarcoplasmic reticulum is represented by J_{RyR} and calcium uptake by $J_{SERCA_{TSR}}/J_{SERCA_{SR}}$, described within the main text. L_e is the rate at which calcium leaks out of the sarcoplasmic reticulum into the myoplasm and t_R is the time constant for calcium diffusion between terminal and bulk myoplasm spaces. The "on" and "off" k rate constants represent binding and dissociation reaction rates between ions involved in calcium buffering ($[CaATP]$; $[CaParv]$) and crossbridge cycling ($[T_0]$; $[CaT]$; $[CaCaT]$; $[D_0]$; $[D_1]$; $[D_2]$).

2. Terminal and Bulk Mitochondrial Calcium Concentrations

Calcium concentration within the bulk and terminal mitochondrial spaces was dependent on the flux through the MCU, NCX, and mPTP (Wacquier et al. 2016), scaled by the volume of the respective sub-compartment:

$$
\frac{d[Ca^{2+}]_{M_x}}{dt} = \frac{1}{V_{M_x}}\Big[f_m\Big(J_{MCU_{M_x}} - J_{NCX_{M_x}} + J_{mPTP_{M_x}}\Big)\Big] \qquad x \in \{TM, M\}
\tag{2.1}
$$

The mitochondrial calcium concentration was additionally scaled by f_m to account for calcium buffering reactions within the mitochondria that reduce the amount of freely available calcium.

3. Oxidative Phosphorylation (OXPHOS)

Mitochondrial NADH was modeled to accumulate as (Wacquier et al. 2016):

$$\frac{d[NADH]_{M_x}}{dt} = J_{PDH_{M_x}} - J_{ETC_{M_x}} + J_{AGC_{M_x}} \quad x \in \{TM, M\} \tag{3.1}$$

acting as the driving force of the electron transport chain in the model within both the terminal and bulk mitochondria spaces. Mitochondrial NAD+ levels were modeled with a conservation equation (Wacquier et al. 2016):

$$[NAD^+]_{M_x} = [NAD]_M^{TOT} - [NADH]_{M_x} \quad x \in \{TM, M\} \tag{3.2}$$

where $[NAD]_M^{TOT}$ is the total concentration of oxidized and reduced NAD ions within the mitochondria, set to 2,970 μM from a computational study in skeletal muscle (Korzeniewski and Zoladz 2001).

4. Proton Leak

A mitochondrial proton leak was included in the model (Wacquier et al. 2016):

$$J_{H,le} = q_9 \Delta\Psi_{M_x} + q_{10} \quad x \in \{TM, M\} \tag{4.1}$$

where q_9 represents the voltage dependence of the leak flux rate and q_{10} represents the rate of the voltage-independent component of the leak. The mitochondrial proton leak couples NADH oxidation by the ETC to ATP synthesis by the F1F0 (Divakaruni and Brand 2011).

5. Myoplasmic ATP

Myoplasmic ATP levels were modeled with dependence on the amount of ATP translocated into the myoplasm via the ANT and the amount of ATP consumed in the myoplasm:

$$\frac{d[ATP]_x}{dt} = \frac{1}{V_x}\left[J_{ANT_{M_x}} - J_{HYD_x} - \tau_{ATP}(ATP_{TM} - ATP_M)\right] - k_{CATP}^{on}[Ca^{2+}]_x[ATP]_x + k_{CATP}^{off}[CaATP]_x - k_{MATP}^{on}[Mg]_x[ATP]_x + k_{MATP}^{off}[MgATP] \tag{5.1}$$

$$x \in \{TM, M\}$$[1]

where the amount of ATP consumed in the terminal myoplasm is represented by $J_{HYD_{TM}}$:

$$J_{HYD_{TM}} = \frac{J_{SERCA_{TSR}}}{2} + k_{HYD}\left(\frac{[ATP]_{TM}}{K_H + [ATP]_{TM}}\right) \tag{5.2}$$

$$x \in \{TM, M\}$$[2]

and in the bulk myoplasm by J_{HYD_M}:

$$J_{HYD_M} = \frac{I_{NaK_T}}{5} + \frac{J_{SERCA_{SR}}}{2} + k_{HYD}\left(\frac{[ATP]_M}{K_H + [ATP]_M}\right) \tag{5.3}$$

$$x \in \{TM, M\}$$[3]

The time constant of ATP diffusion between terminal and bulk myoplasm spaces is represented by τ_{ATP} and V_x is a volumetric parameter. The "on" and "off" k rate constants are given in Senneff and Lowery (2021), representing binding and dissociation reaction rates between calcium and ATP ($[Ca^{2+}]$; $[ATP]$), calcium-bound ATP ($[CaATP]$), magnesium and ATP ($[Mg]$; $[ATP]$), and magnesium-bound ATP ($[MgATP]$).

---

[1] The "$-\tau_{ATP}(ATP_{TM} - ATP_M)$" term becomes $+\tau_{ATP}(ATP_{TM} - ATP_M)$ when $x = M$
[2] The "$-\tau_{ATP}(ATP_{TM} - ATP_M)$" term becomes $+\tau_{ATP}(ATP_{TM} - ATP_M)$ when $x = M$
[3] The "$-\tau_{ATP}(ATP_{TM} - ATP_M)$" term becomes $+\tau_{ATP}(ATP_{TM} - ATP_M)$ when $x = M$
