# Supplementary material for: Computational Model of the Effect of Mitochondrial Dysfunction on Excitation–Contraction Coupling in Skeletal Muscle
Source: Bull Math Biol. 2022 Sep 17;84(11):123. doi: 10.1007/s11538-022-01079-3 (PMC9482608; doi:10.1007/s11538-022-01079-3)
Supplement: Supplementary file 2 — Supplementary file2 (PDF 105 KB) [file 11538_2022_1079_MOESM2_ESM.pdf]

Supplementary Material B

Note: This supplementary material introduces parameters used in the mitochondria model. Model parameters not listed here involved in the model of excitation-contraction coupling are given in Senneff and Lowery (2021). Slow twitch fiber parameters were used in this paper and were not changed in this study from Senneff and Lowery (2021) unless otherwise indicated.

Parameter	Unit	Definition	Value (ST)
a_1	-	Scaling factor between NADH consumption and the change in $\Delta\Psi$	120 (Numerically tuned to achieve basal resting $\Delta\Psi$ of 190 mV)
a_2	-	Scaling factor between ATP production and the change in $\Delta\Psi$	3.43 (Wacquier et al. 2016)
C_p	$\mu M\ mV^{-1}$	Mitochondrial membrane capacitance (divided by F)	1.8 (Wacquier et al. 2016)
f_m	-	Fraction of free over buffer-bound calcium within mitochondria	0.01 (Numerically tuned)
V_{MCU}	$\mu M\ ms^{-1}$	MCU rate constant	0.0215 (Numerically tuned)
K_{trans}	μM	K_d for translocated calcium	19 (Cortassa et al. 2003)
$\Delta\psi^{o}_{MCU}$	mV	Offset $\Delta\Psi$ for MCU	91 (Cortassa et al. 2003)
K_{act}	μM	Activation constant for MCU	0.38 (Cortassa et al. 2003)
L	-	Allosteric constant for MCU conformational changes	130 (Numerically tuned)
n_a	-	Hill coefficient for MCU	2.8 (Cortassa et al. 2003)
V_{NCX}	$\mu M\ ms^{-1}$	NCX rate constant	0.00035 (Wacquier et al. 2016)
$\Delta\Psi^{o}_{NCX}$	mV	Offset $\Delta\Psi$ for NCX	190 (Wacquier et al. 2016)

K_{Na}	mM	Antiporter Na^+ constant	9.4 (Cortassa et al. 2003)
K_{Ca}	μM	Antiporter Ca^{2+} constant	1.1 (Numerically tuned)
V_{mPTP}	$\mu M\ ms^{-1}$	Rate constant of mPTP	0.000008 (Wacquier et al. 2016)
p_3	mV^{-1}	Voltage-dependence coefficient of mPTP	0.075 mV^{-1} (Numerically tuned)
$C_{t_{M_{TM}}}$	μM	Threshold for mPTP high conductance state in the terminal mitochondrial space	1824 μM [1]
$C_{t_{M_M}}$	μM	Threshold for mPTP high conductance state in the bulk mitochondrial space	737 μM [2]
V_{GLY}	$\mu M\ ms^{-1}$	Empirical velocity of glycolysis	0.468 (Korzeniewski et al. 2001)
q_1	-	Michaelis-Menten-like constant for Krebs cycle NAD^+ consumption	1 (Wacquier et al. 2016)
q_2	μM	$S_{0.5}$ value for Krebs cycle activation by Ca^{2+}	0.1 (Wacquier et al. 2016)
$[NAD]_M^{TOT}$	μM	Total concentration of mitochondrial NAD	2970 (Korzeniewski et al. 2001)
V_{AGC}	$\mu M\ ms^{-1}$	Rate constant of AGC (NADH production via malate-aspartate shuttle)	0.025 (Wacquier et al. 2016)
K_{AGC}	μM	Dissociation constant of Ca^{2+} from AGC	0.14 (Wacquier et al. 2016)
p_4	mV^{-1}	Voltage-dependence coefficient of AGC activity	0.01 (Wacquier et al. 2016)

[1] This value was calculated as 2.5 times the peak of the mitochondrial calcium transient in the terminal space in response to a single muscle stimulus, scaled by 19.4, the mitochondrial protein density in muscle cells in mg protein/ml (Williams et al. 2013).
[2] This value was calculated as 2.5 times the peak of the mitochondrial calcium transient in the bulk space response to a single muscle stimulus, scaled by 19.4, the mitochondrial protein density in muscle cells in mg protein/ml (Williams et al. 2013).

V_{ETC}	$\mu M\ ms^{-1}$	Rate constant of NADH oxidation by the ETC	0.764 (Korzeniewski et al. 2001)
q_3	μM	Michaelis-Menten constant for NADH consumption by the ETC	100 (Wacquier et al. 2016)
q_4	mV	Voltage-dependence coefficient of ETC activity	177 (Wacquier et al. 2016)
q_5	mV	Voltage-dependence coefficient of ETC activity	5 (Wacquier et al. 2016)
V_{F1F0}	$\mu M\ ms^{-1}$	Rate constant of the F1F0	3.6 (Numerically tuned)
q_6	μM	ATP inhibition constant of F1F0	10000 (Wacquier et al. 2016)
q_7	mV	Voltage-dependence coefficient of F1F0	190 (Wacquier et al. 2016)
q_8	mV	Voltage-dependence coefficient of F1F0	8.5 (Wacquier et al. 2016)
V_{ANT}	$\mu M\ ms^{-1}$	Rate constant of ANT	8.123 (Wu et al. 2007)
θ	-	ANT parameter	0.35 (Wu et al. 2007)
$k_{m,ADP}$	μM	Michaelis-Menten constant for ANT	3.5 (Wu et al. 2007)
q_9	$\mu M\ ms^{-1}\ mV^{-1}$	Voltage-dependence coefficient of proton leak	0.002 (Wacquier et al. 2016)
q_{10}	$\mu M\ ms^{-1}$	Rate constant of voltage-independent proton leak	-0.03 (Wacquier et al. 2016)
